# Metabolomics Reveals Reasons for the Efficacy of Acupuncture in Migraine Patients: The Role of Anaerobic Glycolysis and Mitochondrial Citrate in Migraine Relief

**DOI:** 10.1007/s43657-024-00205-6

**Published:** 2025-05-04

**Authors:** Zishan Gao, Xianzhong Yan, Rui Wang-Sattler, Marcela Covic, Guang Yu, Feifei Ge, Jia Lin, Qin Chen, Juan Liu, Sapna Sharma, Sophie Molnos, Brigitte Kuehnel, Rory Wilson, Jonathan Adam, Stefan Brandmaier, Shuguang Yu, Fanrong Liang, Christian Gieger

**Affiliations:** 1 Department of Clinical Acupuncture and Moxibustion, Nanjing University of Chinese Medicine, Nanjing, 210029 Jiangsu China; 2 Research Unit of Molecular Epidemiology, Helmholtz Zentrum München, German Research Center for Environmental Health, 85764 Neuherberg, Germany; 3 Institute of Epidemiology, Helmholtz Zentrum München, German Research Center for Environmental Health, 85764 Neuherberg, Germany; 4 Institute for Medical Information Processing, Biometry and Epidemiology, Ludwig-Maximilians University Munich, 81377 Munich, Germany; 5 National Center of Biomedical Analysis, Beijing, 100039 China; 6 Department of Biochemistry and Molecular Biology, School of Medicine & Holistic Integrative Medicine, Nanjing University of Chinese Medicine, Nanjing, 210029 Jiangsu China; 7 Department of Human Anatomy and Histoembryology, School of Medicine & Holistic Integrative Medicine, Nanjing University of Chinese Medicine, Nanjing, 210029 Jiangsu China; 8 Department of Biochemistry and Molecular Biology, West China School of Basic Medical Sciences & Forensic Medicine, Sichuan University, Chengdu, 610041 Sichuan China; 9 The Third Affiliated Hospital of Zhejiang, Chinese Medical University, Hangzhou, 310006 Zhejiang China; 10 Acupuncture & Chronobiology Key Laboratory of Sichuan Province, Chengdu University of Traditional Chinese Medicine, Chengdu, 610075 Sichuan China; 11 Acupuncture and Tuina School/The 3rd Teaching Hospital, Chengdu University of Traditional Chinese Medicine, Chengdu, 610075 Sichuan China

**Keywords:** Migraine, Acupuncture, Metabolomics, Anaerobic glycolysis, Citrate, Lactic acid

## Abstract

Acupuncture is used worldwide to treat migraine, but its scientific mechanism remains unclear. Here, we report a ^1^H NMR metabolomics study involving 40 migraine patients and 10 healthy individuals randomly receiving acupuncture or sham acupuncture, followed by machine learning techniques and functional analysis. We found that acupuncture at acupoints particularly enhanced anaerobic glycolysis and modified mitochondrial function by adjusting the levels of plasma pyruvic acid (*p* = 0.012), lactic acid (*p* = 0.031) and citrate (*p* = 0.00079) at a Bonferroni-corrected level of significance compared to the pre-treatment level of these three metabolites in migraine patients. Therefore, acupuncture supplies energy to migraine patients and relieves migraine attacks. In contrast, we observed that sham acupuncture may partially supply energy to migraine patients through lipid metabolism by changing the levels of plasma lipid (*p* = 0.0012), glycerine (*p* = 0.021), and pyruvic acid (*p* = 0.047) at a Bonferroni-corrected level of significance. The functional network analysis further indicates this different way of supplying energy contributes to the different effects of acupuncture and sham acupuncture. Our findings reveal novel metabolic evidence for the specific effect of acupuncture in relation to sham acupuncture. This metabolic evidence could enlighten a brand new direction into acupuncture analgesia mechanism, which in turn would pose fresh challenges for future acupuncture research.

**Supplementary Information:**

The online version contains supplementary material available at 10.1007/s43657-024-00205-6.

## Introduction

Acupuncture is commonly used for preventing and relieving migraine worldwide (Wells et al. 2011). Understanding the mechanism underlying the efficacy of acupuncture for migraine is key to the acceptance of acupuncture as a valid therapy by both Western doctors and policymakers. Recently, a rich source of clinical evidence has demonstrated the effectiveness and safety of acupuncture for migraine, showing that acupuncture could effectively reduce the intensity of migraine, the frequency of migraine attacks, and the number of migraine days (Linde et al. 2016; Vickers et al. 2012). In contrast to the abundant evidence from those clinical trials, the biological basis for the efficacy of acupuncture in relieving migraines remains unclear.

To date, several experimental studies have shown that acupuncture alleviates migraine by activating a range of biochemicals in peripheral and pain-related central nuclei (Goldman et al. 2010; Zhao et al. 2017a, b). Goldman et al. (2010) found that adenosine and adenosine metabolism mediated the analgesic effect of acupuncture in a mouse model. Zhao et al. (2017a, b) showed that calcitonin gene-related peptide (CGRP), which plays a key role in triggering migraine, was suppressed by electroacupuncture in a rat model of migraine. However, there is no validated biomarker associated with the effects of acupuncture for migraine in clinical studies. The explicit biochemical pathways or mechanisms that address the process of acupuncture relief for migraine are not clear at present. Furthermore, the specific effect of acupuncture compared to sham acupuncture is a long-standing controversial topic. Zhao et al. (2017a, b) demonstrated in a multicenter randomized trial that acupuncture for migraine is more effective than sham acupuncture; others, however, have found no differences (Linde et al. 2005; Li et al. 2012). An individual patient data (IPD) meta-analysis including 20,827 patients confirmed that acupuncture is statistically superior to sham acupuncture for migraine, but the difference in effect size between acupuncture and sham was relatively small (Vickers et al. 2012; Vickers et al. 2014). Due to the absence of explainable biological mechanisms for the specific effects of acupuncture, the acceptance of acupuncture as a referable therapy in migraine management is still debatable (Linde et al. 2017). Hence, two important questions are raised in this paper: first, are there any biomarkers or pathways that provide a possible mechanism for the efficacy of acupuncture in relieving migraine? Second, is there any biological basis attributed to the specific effect of acupuncture compared with sham acupuncture for relieving migraine?

Recently, a large number of studies resumed investigations of the pathogenesis of migraine, emphasizing that abnormalities in energy metabolism and mitochondrial function are the fundamental milestones in the pathophysiology of migraine (Colombo et al. 2014; Lodi et al. 2006; Sparaco et al. 2006). Increasing evidence have revealed that insufficient cerebral glycogen unbalances energy metabolism, leading to inhibition of astroglial mitochondrial respiration and excessive production of free radicals. These metabolic changes attribute to further energy failure in neurons that stimulate cortical spreading depression (CSD) and thus trigger migraine (Finsterer et al. 2018). Moreover, previous studies indicated that the effect of acupuncture may involve multiple pathways and dynamic system changes from genomics to metabolomics (Xu et al. 2012). Metabolomics has been powerfully utilized to reveal potential metabolic biomarkers of acupuncture for hypertension (Yang et al. 2018), functional dyspepsia (Wu et al. 2016) and chronic atrophic gastritis (Luo et al. 2013). As such, it is therefore essential to employ metabolomic approaches to pinpoint crucial biomarkers and elucidate the systemic mechanism of acupuncture's effects for migraine. Our previous study demonstrated that acupuncture could adjust metabolic profiles in an acute migraine rat model (Gao et al. 2014). Nevertheless, challenges from statistical methods for dealing with high-dimensional metabolomic data still limit the use of metabolomics in acupuncture research. Within this context, machine learning techniques, such as the least absolute shrinkage and selection operator (Lasso), are promising tools for reducing high-dimensional data and selecting accurate biomarkers for metabolomics (LeWitt et al. 2017; Menni et al. 2017).

**Fig. 1:**
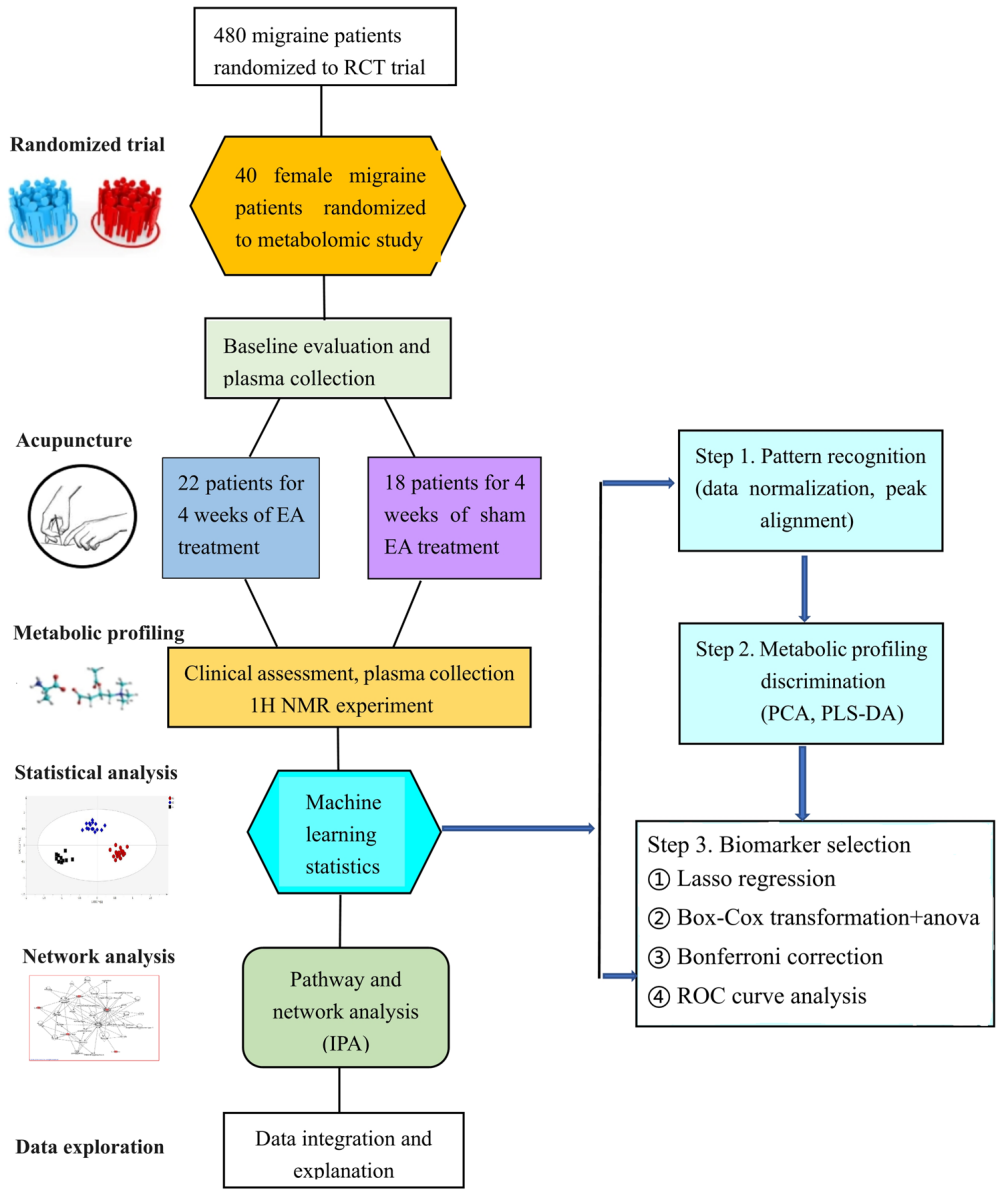
Study design. We conducted a non-targeted metabolomic study of 40 migraine patients and 10 healthy individuals. Plasma was collected before and after EA or Sham EA treatment for ^1^H NMR experiments

To investigate the potential metabolic mechanism for the efficacy of acupuncture relief of migraine, we employed ^1^H- Nuclear magnetic resonance (NMR) metabolomic technology to detect plasma metabolic phenotypes for 40 female migraine patients who randomly received either acupuncture or sham acupuncture from a total of 476 patients in a randomized controlled trial, together with a group of 10 healthy individuals. Using orthogonal signal correction for partial least square discriminate analysis (OPLS-DA), we determined different metabolic profiles among healthy controls and migraine patients treated with acupuncture or sham acupuncture. Next, we conducted Lasso regression to select metabolic biomarkers for discriminating acupuncture and sham acupuncture and validated the selected metabolites using analysis of variance (ANOVA) combined with Box-Cox transformations and Bonferroni corrections. Further, we validated the performance of Bonferroni-corrected significant metabolic biomarkers discriminating migraine patients at baseline, healthy people and migraine patients after acupuncture in a clinical setting by receiver operating characteristic (ROC) curve analysis. Finally, we complemented our analysis with two Bonferroni-corrected significant metabolites and two discriminated metabolic pathways attributed to the specific effect of acupuncture using Ingenuity Pathway Analysis (IPA), revealing a novel metabolic mechanism for the efficacy of acupuncture in relieving migraine (Fig. 1).

## Materials and Methods

### Ethics Approval

The clinical trial protocol was approved by the Ethics Review Committee of the first Teaching Hospital of Chengdu University of Tranditional Chinese Medicine (TCM) and had been published [2007KL-002] (Li et al. 2008). The multicenter randomized trial was registered (clinicaltrials.gov: NCT00599586). All study procedures were designed and conducted in accordance with principles of the Declaration of Helsinki and the Chinese version of the International Conference on Harmonisation—Good Clinical Practice.

### Participants

From April 1, 2008, to August 12, 2009, 476 migraine patients were recruited in the multicenter trial. And the migraine patients included in this metabolomic study were all recruited from a single trial center, the First Teaching Hospital of Chengdu University of Traditional Chinese Medicine, which is one of the clinical centers in the multicentre trial. To be included, the migraine patients had to meet the International Classification of Headache Disorders, 2nd edition (ICHD-II), (Headache Classification Subcommittee of the International Headache Society 2004), as diagnosed by a doctor. The inclusion criteria were as follows: age between 20 and 45 years old; female only; Han nationality; onset of migraines before age 45; more than one year of migraine history and acute migraine attacks two to eight times per month during the previous three months; no use of any prophylactic drug for migraine during the previous two weeks; body mass index (BMI) ranging from 18 to 24; no heart, liver, or kidney disease detected by ultrasound or blood tests as well as other serious organic diseases; willingness to complete 20 acupuncture treatments for four weeks; and ability to provide written informed consent.

We excluded patients who had headache owing to organic disorders (e.g., cerebrovascular disease, vascular malformation, arthritic conditions, arteriosclerosis or hypertension) or patients with psychosis, pregnancy or lactation, allergies, bleeding disorders or serious diseases of the heart, liver, kidney or other organs.

Healthy females without migraine, who had no significant differences in age, gender, or BMI compared to migraine patients, were recruited as the healthy control group.

### Randomization and Interventions

The overall trial approach is shown in Fig. 1. Forty female patients who met the inclusion criteria were randomly assigned to the electroacupuncture group at specific acupoints belonging to the Shaoyang Meridian group (EA) or the electroacupuncture group at non-acupoints (Sham EA) using a central randomization procedure controlled by the Chengdu Good Clinical Practice (GCP) Center. Central randomization was conducted by a GCP center computer. The independent study assistant sent the patient's information to the GCP centre through email or short message service (SMS). Random numbers and group assignments were accordingly generated by a GCP computer and sent back to the study assistant by a feedback email or SMS. Acupuncturists were not involved in patient recruitment in this trial. Patients and outcome assessors were blinded to the randomization. Patients were informed that they would receive one of two types of acupuncture treatment: one was based on traditional Chinese acupuncture theories and the other was based on modern acupuncture theory.

**Fig. 2:**
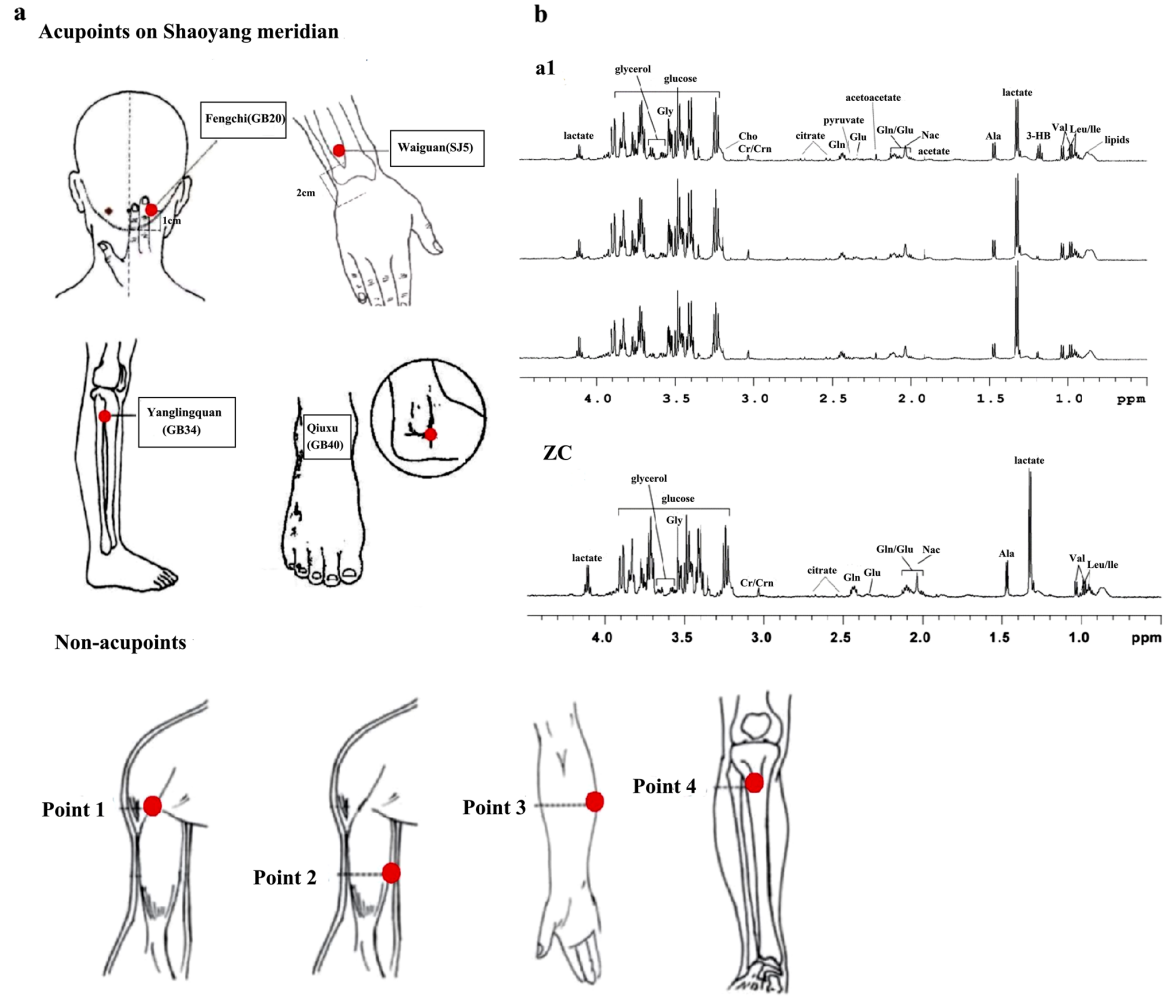
Locations of acupuncture points and typical ^1^H NMR spectra of plasma samples. **a** Locations of acupuncture points at Shaoyang meridian and non-acupoints. **b** Typical ^1^H NMR CPMG spectra of plasma samples. ^1^H NMR experiments were carried out, and Chenomx NMR Suite 4.5 (Chenomx, Calgary, Canada) software was used to identify 22 metabolites measured in a total of 50 plasma samples from 40 migraine patients and 10 healthy controls before and after EA or sham EA treatment (Table 2). a1, migraine patients; ZC, healthy controls; Ala, alanine; Cr/Crn, creatine; Gly, glycine; gln, glutamine; glu, glutamate; Val, valine; 3-HB, 3-hydroxybutyric acid; Leu/lle, isoleucine; Different citrate levels of migraine patients indicate that there is a difference at the citrate level in the spectra

The treatment strategies for selecting acupoints and non-acupoints were developed through consensus among experienced acupuncture practitioners and Western medicine neurologists. These strategies are based on Traditional Chinese Medicine (TCM) theory, meta-analyses, and the results of clinical trials. Acupuncture treatment was performed unilaterally, alternately at specific acupoints at Waiguan (TE5), Yanglingquan (GB34), Qiuxu (GB40), and Fengchi (GB20), which belong to the Shaoyang Meridian of the hand and foot in the EA group (Fig. 2a). Deqi sensation was required for the EA group. In contrast, sham acupuncture treatment was conducted unilaterally, alternately at predefined non-acupoints, including the middle point between the tip of the elbow and the axilla, the middle point between the epicondylus medialis of the humerus and the ulnar side of the wrist on the ulnar side, the edge of the tibia one to 2 cm lateral to the Zusanli (ST36), and the anterior border of the insertion of the deltoid muscle at the junction of the deltoid and biceps muscles in the medial arm (Li et al. 2008) (Fig. 2a). Deqi sensation was not required for the Sham EA group. All acupoints and sham acupoints were punctured by disposable stainless steel needles (0.25 mm × 40 mm; 0.25 mm × 25 mm; Suzhou Hwato Medical Appliance Co., Ltd., 2270202, Suzhou City, China) at a depth of 20–40 mm. In addition to normal puncturing, four auxiliary needles were punctured 2 mm beside every acupoint or non-acupoint with a depth of 2 mm without manual stimulation (Li et al. 2008). Transcutaneous electroacupuncture stimulation was subsequently conducted at the acupoints or sham acupoints for 30 min using a Han's acupoint nerve stimulator (HANS-200, Nanjing, China) after needle insertion. The stimulation frequency was set at 2/15 Hz, and the intensity varied from 0.1 mA to 1 mA, adjusted in accordance with the patient's perception. All acupuncture procedures were conducted in accordance with standards for reporting interventions in clinical trials of acupuncture (STRICA) (MacPherson et al. 2010) and a predefined acupuncture standard operating procedure (SOP) by a licensed acupuncturist with at least five years of clinical acupuncture experience and with a completed postgraduate degree. Acupuncture treatment was performed five times per week according to the patient's convenience, and a total of 20 acupuncture treatments were performed on both the EA and Sham EA groups. The patients were instructed not to take regular medications during acupuncture treatment. However, ibuprofen (300 mg each capsule with sustained release) could be used as rescue medication when severe migraine attacks happened.

To improve and monitor patient compliance, an independent study assistant tracks and records patient information and adherence throughout the treatment sessions. Additionally, an independent manager supervises quality control to ensure patient compliance across the multi-center trial. A specialized quality assessment form is employed to thoroughly evaluate trial procedures, including treatment sessions, dropouts, withdrawals, loss to follow-up, and adverse events. Skilled inspectors conduct these assessments monthly at each participating hospital in the multi-center trial, including our study.

### Outcomes and Sample Collection

The primary outcome for evaluating the frequency of migraine attacks was the number of days with migraines before and after four weeks of treatment. This outcome was recorded in a migraine diary by the migraine patients during the baseline and four-week treatment period. Together with the number of days with migraine, the frequency of migraine and the visual analog scale (VAS) were also recorded by the migraine patients in the same period. The VAS is a validated and reliable instrument for assessing pain severity and alleviation. On this scale, pain is measured by placing a handwritten mark on a 10-cm line representing a continuum between "no pain" (0 cm) and "worst pain" (10 cm). This provides a pain severity rating in centimeters out of 10, such as six out of 10 (or 6/10 cm) (Delgado et al. 2018). Secondary outcomes included the measurements of the frequency and pain intensity of migraine. A total of two blood samples were collected for metabolomics analysis at the start of acupuncture treatment and at the end of four weeks of treatment. Blood samples of healthy control were also collected at the end of four weeks of treatment. All fasting venous blood samples (approximately 5 mL) were collected at approximately 7:30–9:30 am and then stored at – 80 ℃.

### Quality Control for NMR Experiments

To control for potential metabolic confounding factors such as diet, age, geographical, and social factors, we implemented the following quality control procedures for all participants in our metabolomic study:

Inclusion criteria:

The study exclusively includes women aged 20 to 45 who are Han nationals and have been residents of Chengdu and its surrounding areas for at least one year.

All participants were required to match in terms of gender, age, ethnicity, and geographical location.

Exclusion criteria:

Individuals with thyroid disease, diabetes, hyperlipidemia, obesity, or other metabolic and endocrine disorders were excluded.

Participants with any biochemical abnormalities were also excluded.

Patients who had taken medication, consumed alcohol, or experienced a cold or fever within the past 15 days were not included.

Pre-sampling regulations:

All participants, including healthy volunteers, were required to adhere to the following guidelines before sampling:

Diet and caffeine restriction: Avoid consuming beverages or foods containing caffeine (e.g., coffee, chocolate, cola, or tea) for 24 hours before sampling.

Alcohol and preservative restriction: Avoid consuming alcoholic beverages or foods containing preservatives, such as cheese, until the sampling process is completed.

Physical activity restriction: Avoid strenuous physical activity until the sampling process is finished.

### NMR Experiments

^1^H NMR spectra of the plasma samples were collected and analyzed as described in our previous study (Gao et al. 2014). One sample from acupuncture group (migraine patients after acupuncture treatment) and one sample from sham acupuncture group (migraine patients after sham acupuncture treatment) were disqualified for analysis before experiment. Prior to NMR analysis, plasma samples were thawed and centrifuged at 13,000×g, 4 ℃ for 10 min. A total of 300 μL of each supernatant was transferred into a 5 mm NMR tube, mixed with 250 μL of D_2_O for field frequency lock and 50 μL of 3-trimethylsilyl-^2^H_4_-propionic acid sodium salt (TSP) in D_2_O (1 mg/mL) as chemical shift reference. All samples contained a final volume of 600 μL and were vortexed repeatedly.

^1^H NMR data of plasma were acquired on a Varian Inova 600 MHz NMR spectrometer at 27 ℃ using a Carr-Purcell-Meiboom-Gill (CPMG) spin-echo pulse sequence, with a total spin–spin relaxation delay (2 nτ) of 320 ms. Water suppression was achieved by selective saturation of the water peak during the recycle delay (2 s) and mixing time of 150 ms. Free induction decays (FIDs) were collected into 32,000 data points with a spectral width of 8000 Hz over 64 scans. The FIDs were then zero-filled by a factor of two and multiplied by an exponential line-broadening factor of 0.5 Hz prior to Fourier transformation. The diffusion-edited experiments were also carried out with bipolar pulse pair (BPP) -longitudinal eddy current delay (LED) pulse sequence. The gradient amplitude was set at 35.0 G/cm with a diffusion delay of 100 ms. A total of 128 transients and 16,000 data points were collected with a spectral width of 8000 Hz. A line-broadening factor of 1 Hz was applied to FIDs before Fourier transformation (Wang et al. 2004).

All NMR spectra were manually phased and baseline-corrected using Varian NMR system (VNMR) 6.1C software (Varian Inc.). For CPMG spectra, each spectrum over the range of δ 0.4–4.4 was integrated into segments of equal width (0.01 ppm). The spectrum between δ 5.2–8.5 was discarded due to the week signal of aromatic amino acids and the potential lack of association with migraine according to previous studies (Dejong et al. 2007; Harder et al. 2021). For longitudinal eddy current delay (LED) data, each spectrum over the range of δ 0.1–6.0 was segmented into integral regions of equal width (0.01 ppm). The regions containing the residual signals of water (δ 4.6–5.1) were excluded. Interval correlation shifting (icoshift) technique in MATLAB package (version 7.0) were employed to conduct peak alignment. It uses a Fast Fourier Transform (FFT) engine and a greedy algorithm that allows align all spectra to be aligned simultaneously (Savorani et al. 2010). To accommodate these large intensity or concentration variations, the integral values of each spectrum were normalized to a constant sum of all integrals in a spectrum in order to minimize the impacts of concentration variation between samples after ppm segmentation. This total spectrum area (TSA) normalization method performed well in representing NMR spectral intensities (Emwas et al. 2018). Identification of metabolites in the spectra was based on literature and the Chenomx NMR Suite 4.5 (Chenomx, Calgary, Canada) and the Human Metabolome Database (HMDB) (http://www.hmdb.ca/). Specific compounds with multiple peaks were determined by combining the relevant ppm corresponding to the most obvious peaks in the normalized data. Figure 2b identifies and displays the major plasma metabolites. The CPMG pulse sequence was used to emphasize the resonances of small metabolites in plasma, while resonances from macromolecules were attenuated (Fig. 2b). Figure S1 shows diffusion-edited NMR spectra of plasma from each group, displaying the signals of lipid and N-acetylglycoproteins (NAc) groups of glycoproteins. Subtle differences in these spectra were observed by visual examination among groups. Following the identification of metabolites, relative concentration of metabolites could be estimated by the defined area of peak among different group. Further analysis was conducted using supervised learning statistical techniques to discriminate potential metabolic differences among the four groups.

### Statistical Analysis

#### Clinical Statistics

The overall statistical strategy used in this study is shown in Fig. 1. The clinical variables were analyzed using R software (version 3.54). The baseline characteristics and clinical outcomes were based on the intention-to-treat (ITT) population. We excluded cases that only retained the baseline measurement but had missing data for all clinical outcomes. If the data were normally distributed, we planned to use analysis of variance (ANOVA) to detect the differences in numerical variables and performed χ2 tests for categorical variables. If data were not normally distributed, we planned to apply the Kruskal–Wallis test. Continuous data were described as the mean (standard deviation (SD)) with 95% confidence intervals (CIs). Categorical data were illustrated as numbers and percentages. A one-sided test was executed for available data under hypothesis testing in superiority. A *p*-value < 0.05 was defined as statistically significant. Clinical effect sizes were calculated by the R package "effectsize" using the Cohen's d method (Ben et al. 2020). The standardized effect size is defined as the difference in means between two groups divided by the pooled standard deviation. The minimal important difference (MID) for the VAS was calculated and defined by the empirical work from Thorlund et al. (2011), and represents the smallest difference in pain relief that patients regarded as important on average.

### Pattern Recognition

The resulting integral data were transformed into SIMCA-P (version 14.0; Umetrics, Ume˚a, Sweden) for pattern recognition analysis. Before analysis, CPMG data and LED data were Pareto-scaled, in terms of variance stabilization (Worley and Powers 2012). For the purpose of discriminating differences in metabolic profiling among the groups, CPMG data and LED data were both subjected to principal component analysis (PCA). To detect and exclude outliers with abnormal metabolic profile among included migraine patients, PCA was first performed on the normalized ^1^H NMR dataset after Pareto scaling in this study (Mickiewicz et al. 2013). A principal component (PC) score plot and loading plot were used to visualize the data. On the score plot, each point represents an individual sample, and on the loadings plot, each point represents a single NMR spectral region. In the score plot, R^2^Y displayed the proportion of the sum of squares for the selected component, which accounts for the proportion of the variance in the responsible (y) variable explained by the regression model (Fig. S2a, b) (Wang et al. 2004). Furthermore, OPLS-DA was performed to maximize separation and remove variance that was not related to group membership (Worley and Powers 2012). Cross-validated ANOVA (CV-ANOVA) method was also carried out for the cross-validation of OPLS-DA analysis within SIMCA-P software. Metabolites with an impact on differentiation were ranked on the basis of the variables of importance parameter (VIP) method coupled with OPLS-DA, which assesses each variable's relative influence on the model. The metabolites with a value of VIP > 1 were selected and listed as potential biomarkers for the discrimination of model (Menni et al. 2017).

### Biomarker Discovery and Validation

To discover potential metabolite biomarkers for the effects of acupuncture, we developed a strict statistical machine learning strategy that is used for this study (see Fig. S4). Initially, the Shapiro-Wilks test was performed on raw data to check the normal distribution. Following that, OPLS-DA analysis and VIP method are conducted to achieve discrimination between groups and select potential biomarkers. After obtaining the list of potential metabolic biomarkers for migraine and acupuncture from VIP method, Lasso regression was performed on the normalized metabolomic data using the glmnet package in R to identify the most significant metabolites determinants of change for migraine and acupuncture. The overall penalty parameter α was set to one to select those with the highest predicted value of metabolites in glmnet. Tenfold internal cross-validation with cv.glmnet function was applied to validate the regression model and to identify the minimum lambda (λ), the best predict parameter for Lasso regression in the model. Through using this minimum λ yielded the most optimized model, the most relevant metabolites distinguishing migraine, acupuncture and sham acupuncture could be subsequently selected (Friedman et al. 2010). For the validation of potential metabolic biomarkers, ANOVA with Box-Cox transformed method was subsequently performed on these metabolites to obtain the Bonferroni-corrected *p*-value with the Mass package in R (Venables et al. 2002; Blaise et al. 2016). Following the analysis from VIP method, LASSO regression and ANOVA, a venn diagram was generated to determine the overlapping metabolites selected by three aforementioned statistical methods (Fig. S4). The overlap of statistically significant metabolites (*p* < 0.05 after Bonferroni correction) common to all three statistical methods were identified as validated biomarkers of migraine and acupuncture. Finally, receiver operating characteristic (ROC) curve analysis was performed to evaluate the diagnostic abilities of these validated biomarkers for migraine and acupuncture. GraphPad Prism version 7.0 (GraphPad Software, United States) was used to perform this ROC analysis. For the calculated area under the curve (AUC) of the biomarker, an AUC of 0.9–1.0 suggested excellent performance, and 0.8–0.89 indicated good performance, while AUC < 0.6 showed nonsignificant diagnostic performance (Haase-Fielitz et al. 2009). Employing this consistent statistical strategy, we were able to subsequently select and narrow the potential biomarkers from series of metabolites and pinpointed the most validated biomarkers for migraine and acupuncture (Yu et al. 2020).

### Pathway and Network Analysis

Ingenuity Pathway Analysis (IPA) software (IPA build version: 364062 M, content version: 26127183, release date: 2015-12-12, analysis date: 2018-11-30, http://www.ingenuity.com/) (QIAGEN, Redwood City, CA, USA) was used to explore potential targeted pathways and networks related to both migraine and the effect of acupuncture in an unbiased way. The ratios of metabolites between two groups, including healthy controls vs. migraine group, acupuncture group vs. migraine group, acupuncture group vs. sham acupuncture group, were calculated and input into IPA software for pathway analysis of migraine and the efficacy of acupuncture. IPA's Core Analysis module was subsequently used for pathway analysis. Fisher's exact test was used to produce a *p* -value to determine the probability that the link between the metabolites and the canonical pathway was explained only by chance. Canonical pathways with a *p*-value < 0.05 after Bonferroni correction were regarded as statistically significant pathways contributing to the pathology of migraine and the potential effect of acupuncture. Based on the 'master' network, which was developed from the Ingenuity Knowledge Base, a causal network was established to reflect observed cause-effect relationships among chemicals, protein families, complexes and biological processes. The network score was calculated using the hypergeometric distribution, with Fisher's exact test at the right tail yielding the negative logarithm of the significant threshold. Z-score > 2 was defined as the threshold of significant activation for network analysis, disease, and function, while Z-score < 2 was defined as the threshold of significant inhibition. The score of the networks also reflects the probability of molecules gathering in this network. When the number of metabolites gathering in this network increases, the score will also increase (Krämer et al. 2014; Kriebel et al. 2016). Following the IPA analysis, the summary of significant pathways and important networks were presented in the supplementary Fig. S3.

## Results

### Baseline Characteristics and Clinical Effects of Acupuncture

A total of 476 migraine patients were included in the multicenter trial (Li et al. 2012), and 40 eligible female patients were enrolled in the four week baseline period and randomized into the true electroacupuncture (EA) and sham electroacupuncture groups (Sham EA) (22 in the EA group, 18 in the Sham EA group) of the metabolomic study. These 40 patients completed both the four week baseline assessment and the four week EA or Sham EA treatment (Fig. 1). Of the 40 patients, 39 met the criteria for migraine without aura, while one patient with migraine with aura was recruited. However, this patient dropped out at the end of the electroacupuncture treatment and was not included in the statistical analysis. The baseline characteristics and clinical outcomes were based on the intention-to-treat (ITT) population in this study. We excluded cases that had only baseline measurements but were missing data for all clinical outcomes. No significant difference between the two groups was found for any demographic characteristics of all included patients, including age, sex, height, weight, or disease status of the patients, such as duration of disease, number of days with migraine, and VAS that assesses pain severity (Table 1, Table S1). A healthy control group consisting of 10 females without migraine, who were matched in terms of age, gender, and BMI with the included migraine patients, was also recruited.

**Table 1:** Clinical outcome measurements between the EA and Sham EA groups before and after treatment

Outcome	EA group (n = 22^#^)	Sham EA group (n = 18^#^)	T value (EA vs Sham)	*P* value (EA vs Sham)	Effect size (EA vs Sham)
Number of days with migraine					
Baseline, Mean, (sd), (95% CI)	7.53 (5.42) (4.25–10.81)	6.46 (3.46) (4.54–8.38)	0.61	0.54	
End of treatment, mean, (sd), (95% CI)	4.92 (5.13) (1.81–8.02)	4.57 (2.73) (2.99–6.15)	0.21	0.82	0.086 (− 0.71 to 0.88)
Difference in means End of treatment- baseline, mean, (sd), (95% CI)	2.61 (2.87) (0.87–4.35)	2.07 (3.64) (− 0.03 to 4.17)	0.43	0.66	0.16
T value	3.28	2.12			
* P* value	0.0065**	0.053			
Effect size	0.50 (0.17–0.82)	0.65 (− 0.04 to 1.35)			
Frequency of migraines					
Baseline, Mean, (sd), (95% CI)	4.69 (2.05) (3.44–5.93)	4.26 (2.28) (3.00–5.53)	0.51	0.60	
End of treatment, Mean, (sd), (95% CI)	3.15 (2.26) (1.78–4.52)	3.57 (1.45) (2.73–4.41)	-0.56	0.57	0.22 (-0.57–1.02)
End of treatment-baseline, Mean, (sd), (95% CI)	1.53 (2.40) (0.08–2.98)	0.71 (2.78) (− 0.89 to 2.32)	0.82	0.41	0.32
T value	2.30	0.95			
* P* value	0.039*	0.35			
Effect size	0.71 (7.03e-05–1.42)	0.36 (− 0.44 to 1.17)			
VAS score					
Baseline, Mean, (sd), (95% CI)	4.11 (1.32) (3.31–4.91)	4.49 (1.60) (3.63–5.35)	− 0.70	0.48	
End of treatment, mean,(sd), (95% CI)	3.24 (1.53) (2.31–4.17)	4.29 (1.95) (3.24–5.33)	− 1.60	0.12	0.58 (− 0.19 to 1.36)
End of treatment-baseline, Mean, (sd), (95% CI)	0.86 (1.99) (− 0.33 to 2.07)	0.25 (1.74) (− 0.71 to 1.22)	0.85	0.40	0.33
T value	1.81	0.56			
* P* value	0.047*	0.573			
Effect size	0.61 (− 0.26 to 1.47)	0.14 (− 0.36 to 0.64)			

*p < *0.05*

**p < *0.01*

#The baseline characteristics and clinical outcomes were based on the intention-to-treat (ITT) population. We omitted the cases that retained only the baseline measurement but had missing data for all clinical outcomes

After four weeks of EA treatment, patients in the EA group showed a significant reduction in days (*p* = 0.006) and frequency (*p* = 0.039) of migraines, as well as a decrease in VAS scores for pain intensity (*p* = 0.047) compared to the baseline period (Table 1). In contrast, patients in the Sham EA group also experienced a decrease in all three symptoms, but this decrease was not statistically significant (*p* > 0.05) (Table 1). However, there was no significant difference between the EA group and the Sham EA group in alleviating migraine (Table 1). Our results were consistent with the multicentre trial on acupuncture for migraine (Li et al. 2012). Since statistical significance alone may not fully explain the magnitude of acupuncture's clinical effects, we calculated the effect size to further validate its efficacy in a clinical setting (Zhao et al. 2017a, b).

After four weeks of treatment, the effect size measured by Cohen's d in the EA group compared to the baseline period was 0.50 (95% CI 0.17–0.82) for the reduction in the number of migraine days, 0.71 (95% CI 7.03e-05–1.41) for the decrease in the frequency of migraines and 0.61 (95% CI − 0.26 to 1.47) for the decrease in VAS scores (Table 1). In the context of our study, these effect sizes beyond 0.5 in the EA group manifested that the effect sizes in the EA group are, on average, 0.5 standard deviations greater for relieving those symptoms of migraine than those during the baseline period (Ben et al. 2020). However, the effect sizes in the Sham EA group compared to the baseline period were 0.65 (95% CI − 0.042 to 1.34) for the reduction in the number of migraine days, 0.36 (95% CI − 0.44 to 1.17) for the decrease in frequency of migraines and 0.14 (95% CI − 0.36 to 0.63) for the decrease in VAS scores (Table 1). These results of effect size were partly in accordance with the large IPD meta-analysis (Vickers et al. 2012), although they were not statistically significant. Specifically, the effect size in the EA group was 0.585 (95% CI − 0.19 to 1.36) compared to the Sham EA group, which means that the EA group is, on average, 0.585 standard deviations greater for decreasing VAS scores than the Sham EA group (Table 1).

### Metabolic Discrimination Between Migraine and Healthy Controls

**Fig. 3:**
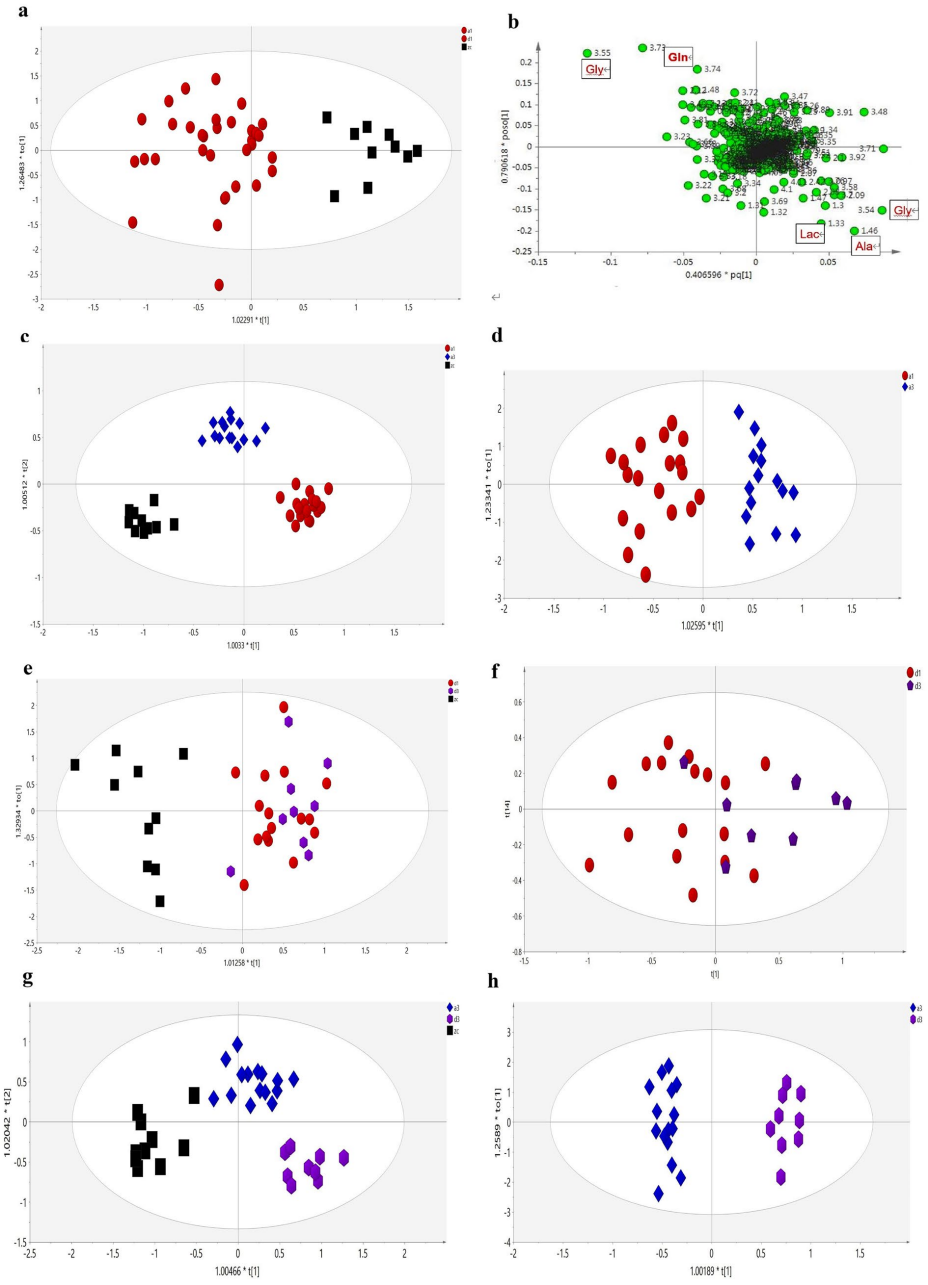
Clear separation of metabolic profiles among groups. OPLS-DA analysis for CPMG data manifested clear separation among migraine patients (red dots), healthy controls (black boxes), migraine patients after four weeks of EA treatment (blue diamonds), and migraine patients after four weeks of sham EA treatment (purple stars). t[1] and t[2] represent the first and second components in the OPLS-DA result, respectively. The missing samples from the EA group and Sham EA group on the score plots were excluded due to the outlier and drop out. **a** Clear separation of metabolic profiling was achieved between migraine (red dots, n = 40) and healthy control (black boxes, n = 10) groups. **b** Corresponding loading plots showing metabolites that may influence the separation for (a). Gly, glycine; Gln, glutamine; Lac, lactic acid; Ala, alanine. **c** The separation of metabolic profiling showed that EA treatment (blue diamonds, n = 22) reversed the change in metabolic profiling in migraine patients (red dots, n = 22) compared with healthy controls (black boxes, n = 10) (Table 3). **d** The results showed a clear discrimination in metabolic profiling between migraine patient after EA treatment (blue diamonds, n = 22) and migraine patients before EA treatment (red dots, n = 22) (Table 3). **e** The results showed that migraine patient after sham EA treatment (purple stars, n = 18) could not restore the change of metabolic profiling in migraine patient before sham EA treatment (red dots, n = 18) compared with healthy controls (black boxes, n = 10) (Table 4). **f** The result showed metabolic profiling of migraine patient before sham EA treatment (red dots, n = 18) could not be discriminated with migraine patient after sham EA treatment (purple stars, n = 10) (Table 4). **g** The profiling indicated that the metabolic profiling of migraine patients after EA treatment (blue diamonds) was closer to that of healthy controls (black boxes) compared to migraine patients after sham EA treatment (purple stars). **h** Clear separation of metabolic profiling was discriminated between EA treatment (blue diamonds) and sham EA treatment (purple stars) (Table 5)

**Fig. 4:**
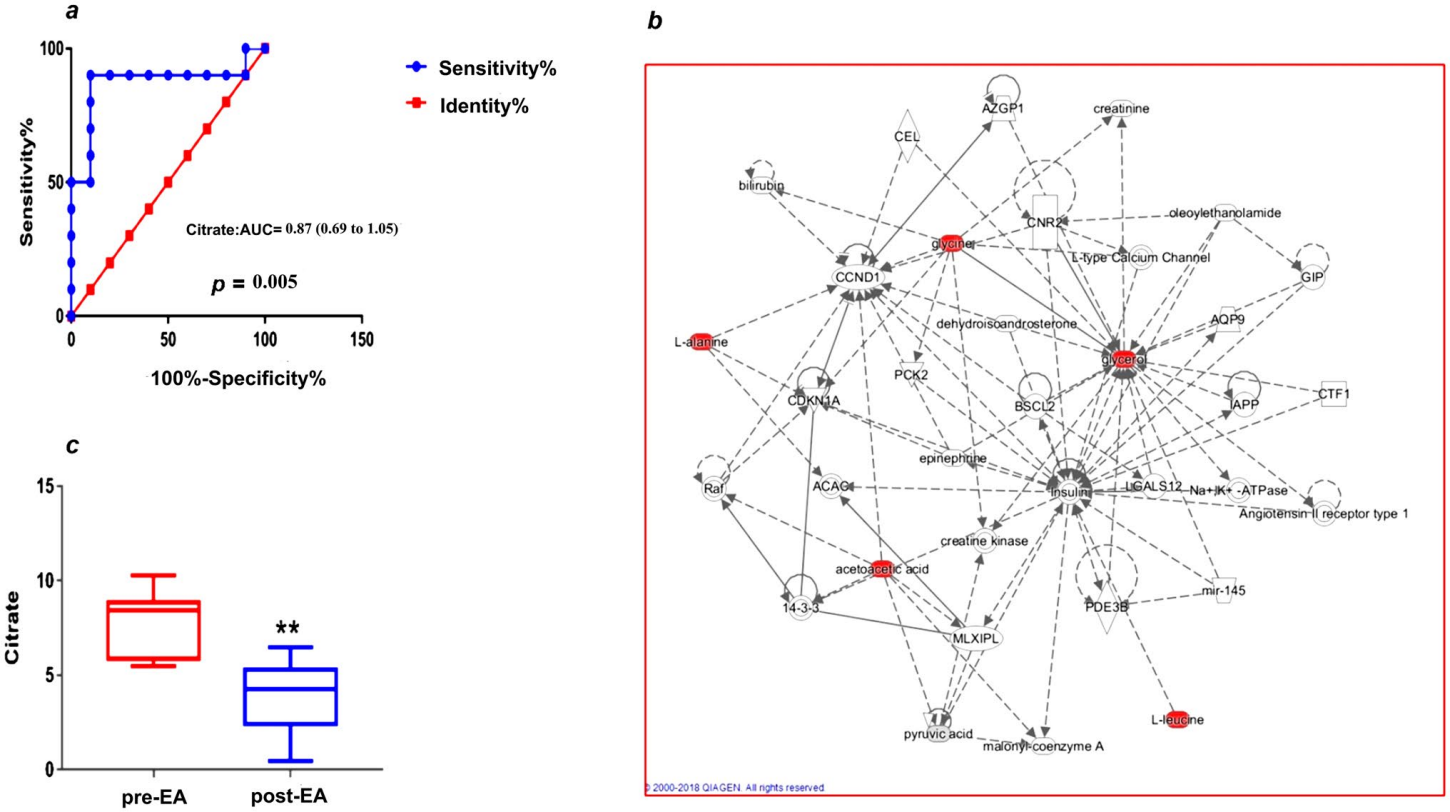
Identification of significant metabolites for the clinical efficacy of acupuncture. We conducted receiver operating characteristic (ROC) curve analysis to validate the significance of potential biomarkers for migraine and acupuncture. **a** ROC analysis showed that citrate could significantly discriminate the migraine and control groups and thus might be a potential diagnostic biomarker (AUC = 0.87) for migraine diagnosis. **b** Citrate was significantly decreased (*p* = 0.00079) after EA treatment. **c** Employing IPA network analysis, we found that glycerine (glycerol), glycine, acetone, alanine and leucine might be important metabolites for the metabolic network of EA vs sham EA. Glycerine (glycerol), which is located near the centre of the metabolic network of EA vs sham EA, may be the key metabolite for the efficacy of EA and sham EA (**c**) (Table 5)

We employed ^1^H NMR techniques and Chenomx NMR Suite 4.5 (Chenomx, Calgary, Canada) to identify 22 metabolites that were measured in a total of 50 plasma samples from migraine patients in the baseline period (MA, n = 40) and healthy controls (n = 10) (Fig. 2 and Table 2). After excluding outliers with abnormal metabolic profiles and baseline values among included migraine patients using PCA analysis, we first performed OPLS-DA analysis on the normalized CPMG NMR data to distinguish metabolic profiles of migraine patients in the baseline period from those of healthy controls. Using SIMICA-P statistical software, OPLS-DA analysis demonstrated a distinct separation between the MA group and healthy controls (Fig. 3a; x-axis; *R*^2^*Y* = 75.8%). In the above score plot, R^2^Y manifested the proportion of the variance in the y variable explained by the regression model (Wang et al. 2014). Thus, the separation in the plot manifested a crucial metabolic phenotype difference between migraine patients and healthy controls. Correspondingly, the loading plot showed the information of the possible metabolites separating the metabolic profiling (Fig. 3b), such as glycine, glutamine, and alanine. After we identified the distinct metabolites in the loading plot, we subsequently conducted Lasso regression, ANOVA combined with Box-Cox transformations and Bonferroni correction on the CPMG dataset to further explore potential metabolic biomarkers separating the profiles of migraine patients and healthy controls. The dominant metabolites that discriminate these two groups are presented in Table 2. After Bonferroni correction, we found a significant increase in five plasma metabolites, including glycerine (*p* = 0.00085), choline (*p* = 0.004), citrate (*p* = 0.016), pyruvic acid (*p* = 0.049) and glutamine (*p* = 0.0017), and a significant decrease in three plasma metabolites, including glycine (*p* = 0.0017), alanine (*p* = 0.002), and lipid (*p* = 0.030) in the MA group compared to healthy controls. We further conducted ROC curve analysis to validate the clinical importance of these biomarkers. The result of the ROC curve analysis showed that citrate performs well in differentiating between migraine patients and healthy persons (AUC = 0.87, *p* = 0.005) (Fig. 4c).

**Table 2:** Changes in plasma metabolites in CPMG NMR spectra between healthy controls and migraine patients

Metabolism	Metabolites	Peak Regions	Con (n = 10) Mean (sd)	Mig (n = 40) Mean (sd)	Direction of effect Mig vs Con	Adj*p* Mig vs Con
Glucose	Glucose	3.89	173.93 (12.32)	171.37 (12.02)	↓	0.56
metabolism	Lactic acid	1.32	276.77 (52.99)	266.91 (55.56)	↓	0.62
	Nac	2.03–2.04	168.04 (12.44)	161.05 (13.89)	↓	0.16
	3-HB	1.19–1.20	35.50 (31.16)	41.81 (23.59)	↑	0.53
	Acetoacetate	2.22	16.24 (14.15)	19.64 (12.12)	↑	0.49
	Citrate	2.68–2.70	3.25 (2.53)	5.73 (2.79)	↑↑	0.016*
	Pyruvic acid	2.38	2.25 (1.63)	3.47 (1.63)	↑↑	0.049*
	Acetate	1.91	9.16 (3.82)	11.33 (5.18)	↑	0.23
Lipid	Lipid	1.3	54.38 (9.31)	42.78 (15.32)	↓↓	0.030*
metabolism	Glycerine	3.64–3.66	70.28 (11.46)	86.66 (12.84)	↑↑	0.00085**
	Ptdcho	3.24	282.6 (16.49)	289.60 (12.72)	↑	0.16
	Choline	3.22–3.23	256.83 (21.51)	284.53 (26.08)	↑↑	0.00403**
	LDL_VLDL	0.86–0.88	158.52 (21.80)	164.31 (36.37)	↑	0.63
Amino acid	Glycine	3.54	239.82 (14.61)	212.26 (24.63)	↓↓	0.0017**
metabolism	Alanine	1.46	83.34 (18.50)	61.05 (18.75)	↓↓	0.0020**
	Glutamate	2.35–2.36	24.74 (5.92)	25.05 (7.41)	↑	0.90
	Valine	1.03–1.04	104.63 (12.72)	103.51 (16.66)	↓	0.85
	Leu_lle	0.97–0.99	145.68 (16.16)	143.82 (21.32)	↓	0.81
	Glutamine	2.43–2.44	94.35 (14.19)	88.95 (15.23)	↓	0.32
	Cre/Crn	3.03–3.04	35.23 (6.39)	38.81 (7.72)	↑	0.19
	Isoleucine	0.94	33.51 (3.71)	31.71 (4.03)	↓	0.21
	Glutamine	2.11–2.12	68.37 (11.86)	81.73 (17.70)	↑↑	0.0017**

The up or down arrow (↑/↓) indicates whether the metabolite showed a signal increase or decrease, respectively

*Con* healthy control, *Mig* migraine patients from the EA and Sham EA groups before treatment, *adjp p* value after Bonferroni correction

*p < *0.05*

**p < *0.01*

Moreover, we conducted OPLS-DA analysis on the ^1^H NMR LED dataset, and the LED results also illustrated a similar separation between the MA group and the healthy control group (Fig. S2c and Table S2; *R*^2^*Y* = 99.7%). Accordingly, we found a significant decrease in plasma N-acetyl glycoproteins (NAc) (*p* = 0.00082) and a significant increase in plasma phosphatidylcholine (Ptdcho) (*p* = 0.035) in the MA group compared to the healthy control group after Bonferroni correction (Table S2). These significantly changed metabolites found in the CPMG and LED results were selected as potential biomarkers for the metabolic features of migraine in the clinical setting.

### Acupuncture Reversing Metabolic Profiling and Plasma Citrate of Migraine

The main objective of our study was to investigate the possible metabolic mechanism for the effectiveness of acupuncture treatment for migraine. Thus, after demonstrating the metabolic profiling of migraine patients, we next explored whether acupuncture changed the metabolites of migraine patients after 20 sessions of EA treatment. Similarly, we performed OPLS-DA analysis on metabolomic data to compare migraine patients during the baseline period (MA; n = 22) and migraine patients after acupuncture treatment (EA; n = 22). To determine the trend of acupuncture altering metabolites, we also conducted an OPLS-DA analysis on the metabolomic data of the two aforementioned groups and 10 healthy controls (n = 10). After 20 sessions of EA at Shaoyang Meridian acupoints over four weeks, we observed that the metabolic profiles of the EA group were close to those of the healthy control group but differed from those of the group at baseline (Fig. 3c; *R*^2^*Y* = 94.5%), showing that EA restores the metabolic profiles of migraine similar to those of healthy controls. We further achieved a clear discrimination in metabolic profiling between migraine patients during the baseline period and migraine patients after acupuncture treatment (Fig. 3d; *R*^2^*Y* = 85.2%). Notably, we demonstrated a significant decrease in the plasma levels of citrate (*p* = 0.00079) and pyruvic acid (*p* = 0.012) in the EA group compared to the MA group after Bonferroni correction (Table 3). This result indicated that EA relieves migraine by altering the levels of plasma citrate and pyruvic acid in the clinical setting. Additionally, we detected a significant increase in plasma lactic acid (*p* = 0.031) and a significant decrease in plasma acetoacetate (*p* = 0.025) in the EA group compare to the group at baseline before EA treatment. Further, we found that glycerine (*p* = 0.00016) was significantly elevated in the EA group compared to the healthy control (Table 3). These significantly changed metabolites may serve as biomarkers for EA's efficacy in relieving migraine. The LED data also showed a clear differentiation between migraine patients during the baseline period and migraine patients after acupuncture treatment (Fig. S2e; *R*^2^*Y* = 99%). A significant decrease in NAc (*p* = 0.00073) was found in the EA group compared with the healthy control group after Bonferroni correction (Table S3). Taken together, these data demonstrated that EA could enhance anaerobic glycolysis by lowering the plasma levels of citrate and pyruvic acid while increasing the plasma level of lactic acid. These regulations in energy metabolism and the tricarboxylic acid cycle (TCA) cycle may be the basis of how acupuncture restores metabolic profiles and relieves migraine in a clinical setting.

**Table 3:** Changes in plasma metabolites in CPMG NMR spectra before and after four weeks of EA treatment in migraine patients

Metabolism	Metabolites	Con (n = 10) Mean (sd)	Mig (n = 22) Mean (sd)	Direction of effect Con vs Mig	Adj*p* Mig vs Con	EA Mean (sd)	Direction of effect EA vs Con	Adj*p* EA vs Con	Direction of effect EA vs Mig	Adj*p* EA vs Mig
Glucose metabolism	Glucose	173.93 (12.32)	166.53 (12.35)	↓	0.33	171.13 (13.78)	↓	0.85	↑	0.58
	Lactic acid	276.76 (53.0)	269.56 (55.83)	↓	0.95	327.16 (71.12)	↑	0.12	↑↑	0.031*
	Nac	168.04 (12.43)	157.42 (14.98)	↓	0.18	160.29 (15.56)	↓	0.40	↑	0.85
	3-HB	35.50 (31.16)	49.46 (29.14)	↑	0.34	30.36 (14.42)	↓	0.89	↓	0.097
	Acetoacetate	16.24 (14.15)	21.11 (15.45)	↑	0.58	9.26 (6.23)	↓	0.35	↓↓	0.025*
	Citrate	3.25 (2.53)	7.24 (2.37)	↑↑	0.00019**	4.02 (1.80)	↑	0.65	↓↓	0.00079**
	Pyruvic_acid	2.25 (1.63)	4.10 (1.93)	↑↑	0.02*	2.32 (1.36)	-	0.99	↓↓	0.012*
	Acetate	9.17 (3.82)	11.34 (5.60)	↑	0.45	10.17 (3.40)	↑	0.84		0.76
Lipid metabolism	Lipid	54.38 (9.31)	46.61 (17.20)	↓	0.37	40.64 (14.21)	↓	0.06	↓	0.49
	Glycerine	70.28 (11.46)	89.79 (12.67)	↑↑	0.00056**	92.26 (11.55)	↑↑	0.00016**	↑	0.83
	Ptdcho	282.60 (16.50)	288.13 (11.53)	↑	0.63	283.87 (17.76)	↓	0.98	↓	0.71
	Choline	256.83 (21.51)	289.91 (27.11)	↑↑	0.0053**	283.68 (24.50)	↑↑	0.03*	↓	0.76
	LDL_VLDL	158.52 (21.80)	162.88 (36.51)	↑	0.95	156.25 (39.11)	↓	0.98	↓	0.85
Amino acid metabolism	Glycine	239.82 (14.61)	218.25 (28.46)	↓	0.13	217.75 (31.54)	↓	0.13	/	0.99
	Alanine	83.34 (18.50)	64.71 (22.65)	↓	0.07	67.57 (20.08)	↓	0.17	↑	0.91
	Glutamate	24.75 (5.92)	25.32 (8.54)	↑	0.98	26.42 (8.87)	↑	0.87	↑	0.92
	Valine	104.63 (12.72)	99.78 (17.20)	↓	0.67	99.17 (12.03)	↓	0.63	/	0.99
	Leu_lle	145.68 (16.16)	138.51 (22.07)	↓	0.59	139.1 (15.84)	↓	0.67	/	0.99
	Glutamine	94.35 (14.18)	92.04 (18.14)	↓	0.92	83.90 (13.86)	↓	0.25	↓	0.33
	Cre_Crn	35.23 (6.39)	42.09 (8.33)	↑	0.08	36.30 (7.95)	↑	0.94	↓	0.10
	Isoleucine	33.51 (3.71)	31.14 (4.86)	↓	0.33	32.14 (3.41)	↓	0.70	↑	0.77
	Glutamine	68.37 (11.86)	79.16 (21.54)	↑↑	0.045*	73.89 (13.72)	↑	0.24	↓	0.65

The up or down arrow (↑/↓) indicates whether the metabolite showed a signal increase or decrease, respectively

*Mig*, migraine patients in EA group before treatment, *EA* migraine patients in the EA group after four weeks of EA treatment, *Con* healthy control; adj*p*, *p* value after Bonferroni correction

*p < *0.05*

**p < *0.01*

### Metabolic Basis for the Specific Effect of Acupuncture

The second objective of our study was to identify the metabolic basis for the specific effect of acupuncture in comparison with sham acupuncture. To address this question, we next performed an OPLS-DA analysis on both CPMG and LED data comparing the true acupuncture group (EA, n = 22) with the sham acupuncture group (Sham EA, n = 18) groups. To distinguish the trend of acupuncture adjusting metabolic profiles, OPLS-DA analysis was performed on metabolomic data from healthy controls (n = 10), true acupuncture (EA, n = 22), and sham acupuncture (Sham EA, n = 18) groups. After 20 sessions of Sham EA treatment at non-acupoints for four weeks, we found that the metabolic profile of the Sham EA group (patients in the Sham EA group after treatment; n = 18) was distinct from the healthy controls but similar to the MA group (patients in the Sham EA group before treatment; n = 18) (Fig. 3e; *R*^2^*Y* = 48.9%). Moreover, the OPLS-DA analysis failed to differentiate MA group and Sham EA group (Fig. 3f; *R*^2^X = 89.9%). In addition, the OPLS-DA results also demonstrated an important discrimination in metabolic profiling among EA, Sham EA and healthy controls (Fig. 3g; *R*^2^*Y* = 88.5%), which illustrated that the metabolic profiling of the EA group was close to the healthy controls in relation to the Sham EA group. The difference in the metabolic profiles suggested that EA treatment, not Sham EA treatment, could reverse the metabolic profiles of migraine patients to healthy controls. Further, the OPLS-DA results showed a clear distinction in metabolic profiles between the EA group and Sham EA group (Fig. 3f; *R*^2^*Y* = 94.8%), which indicates a discriminated metabolic phenotype between true acupuncture and sham acupuncture. The cross-validation for the OPLS-DA analysis of the aforementioned groups was conducted using CV-ANOVA within SIMICA-P software, and the results are provided in Table S6.

Corresponding to the OPLS-DA analysis, after Bonferroni correction, ANOVA results showed a significant decrease in pyruvic acid (*p* = 0.047) in the Sham EA group compared to the MA group (Table 4). Similarly, we found a significant decrease in lipid (*p* = 0.0012), glycine (*p* = 0.000013), and alanine (*p* = 0.0016) as well as a significant increase in glycerine (*p* = 0.021) and glutamine (*p* = 0.000013) in the Sham EA group compared with the healthy control (Table 4). These metabolite changes induced by Sham EA reflect the potential metabolic basis for the effect of sham acupuncture on migraine. Specifically, by using Lasso regression analysis, glycine, glycerine, alanine, 3-hydroxybutyric acid (3-HB), isoleucine (leu_lle), low density lipoprotein_very low density lipoprotein (LDL_VLDL), acetoacetate and glutamine were further selected as potential biomarkers for the discrimination of EA and Sham EA (Table 5). Notably, we found that glutamine (*p* = 0.0098) was significantly decreased in the EA group compared to the Sham EA group (Table 5). Combined, these data demonstrate that there is a distinct metabolic difference between EA and Sham EA. These metabolic differences, which can be caused by plasma changes in glycine, glycerine, alanine, 3-HB, leu_lle, LDL_VLDL, acetoacetate, and glutamine, may provide potential metabolic basis for the specific effects of acupuncture.

**Table 4:** Changes in plasma metabolites in CPMG NMR spectra before and after four weeks of sham acupuncture treatment in migraine patients

Metabolism	Metabolites	Con Mean (sd) (n = 10)	Mig Mean (sd) (n = 18)	Direction of effect Con vs Mig	Adj*p* Mig vs Con	Sham EA Mean (sd)	Direction of effect Sham EA vs Con	Adj*p* Sham EA vs Con	Direction of effect Sham EA vs Mig	Adj*p* Sham EA vs Mig
Glucose metabolism	Glucose	173.94 (12.32)	176.51 (9.54)	↑	0.79	173.66 (8.28)	–	0.99	↓	0.79
	Lactic acid	276.77 (52.99)	264.10 (56.97)	↓	0.85	285.55 (89.80)	↑	0.99	↑	0.80
	Nac	168.04 (12.43)	164.91 (11.88)	↓	0.79	159.32 (9.15)	↓	0.25	↓	0.49
	3-HB	35.50 (31.16)	33.68 (12.07)	↓	0.88	27.48 (14.54)	↓	0.82	↓	0.50
	Acetoacetate	16.24 (14.15)	18.08 (7.32)	↑	0.54	16.72 (12.50)	-	0.97	↓	0.71
	Citrate	3.25 (2.53)	4.14 (2.31)	↑	0.50	4.26 (2.16)	↑	0.57	/	0.99
	Pyruvic acid	2.25 (1.63)	2.81 (0.86)	↑	0.29	1.83 (1.22)	↓	0.64	↓↓	0.047**
	Acetate	9.16 (3.82)	11.32 (4.88)	↑	0.36	11.28 (2.89)	↑	0.35	/	0.97
Lipid metabolism	Lipid	54.38 (9.31)	38.71 (12.27)	↓↓	0.011*	32.80 (12.12)	↓↓	0.0012**	↓	0.39
	Glycerine	70.28 (11.46)	83.33 (12.54)	↑↑	0.019**	84.82 (9.98)	↑↑	0.021*	↑	0.93
	Ptdcho	282.60 (16.49)	291.15 (14.08)	↑	0.30	286.37 (12.20)	↑	0.82	↓	0.71
	Choline	256.83 (21.51)	278.81 (24.46)	↑	0.12	285.47 (38.50)	↑	0.08	↑	0.88
	LDL_VLDL	158.52 (21.81)	165.83 (37.35)	↑	0.92	171.78 (36.20)	↑	0.73	↑	0.89
Amino acid metabolism	Glycine	239.82 (14.61)	205.91 (18.61)	↓↓	9.98498E-05**	196.28 (17.62)	↓	1.35E-05**	↓	0.34
	Alanine	83.34 (18.50)	57.16 (13.10)	↓↓	0.0014**	55.11 (17.37)	↓↓	0.0016**	↓	0.87
	Glutamate	24.75 (5.92)	24.76 (6.27)	–	0.99	24.04 (4.51)	-	0.98	–	0.98
	Valine	104.63 (12.72)	107.46 (15.62)	↑	0.91	108.07 (15.70)	↑	0.89	–	0.99
	Leu_lle	145.69 (16.16)	149.46 (19.59)	↑	0.89	152.31 (20.70)	↑	0.75	↑	0.93
	Glutamine	94.35 (14.19)	85.67 (11.02)	↓	0.29	85.18 (15.72)	↓	0.29	–	0.98
	Cre_Crn	35.24 (6.39)	35.33 (5.31)	–	0.99	38.33 (7.32)	↑	0.56	↑	0.54
	Isoleucine	33.51 (3.71)	32.33 (2.95)	↓	0.69	31.64 (3.86)	↓	0.44	↓	0.85
	Glutamine	68.37 (11.86)	84.47 (12.57)	↑↑	5.30E-05**	88.57 (14.04)	↑↑	1.36E-05 **	↑	0.46

The up or down arrow (↑/↓) indicates whether the metabolite showed a signal increase or decrease, respectively

*Mig* migraine patients in sham EA group before treatment, *Sham EA* migraine patients in the sham EA group after four weeks of sham EA treatment, *Con* healthy control, *adjp p* value after Bonferroni correction

*p < *0.05*

**p < *0.01*

**Table 5:** Changes in plasma metabolites in CPMG NMR spectra between EA and sham EA treatment

Metabolism	Metabolites	EA mean(sd)	Sham EA mean(sd)	Direction of effect EA vs Sham EA	Adj*p* EA vs Sham EA	Coefficient of Lasso EA vs Sham EA
Glucose metabolism	Glucose	171.13 (13.78)	173.67 (8.28)	↑	0.64	n.a
	Lactic acid	327.17 (71.12)	285.55 (89.88)	↑	0.23	n.a
	Nac	160.29 (15.56)	159.32 (9.15)	↓	0.85	n.a
	3-HB	30.36 (14.42)	27.48 (14.54)	↓	0.64	− 0.74
	Acetoacetate	9.26 (6.23)	16.72 (12.50)	↑	0.06	1.06
	Citrate	4.02 (1.81)	4.26 (2.16)	–	0.72	n.a
	Pyruvic acid	2.32 (1.36)	1.83 (1.22)	↓	0.35	n.a
	Acetate	10.17 (3.40)	11.29 (2.90)	↑	0.43	n.a
Lipid metabolism	Lipid	40.64 (14.22)	32.80 (12.13)	↓	0.18	n.a
	Glycerine	92.26 (11.56)	84.824 (9.98)	↓	0.12	− 0.38
	Ptdcho	283.87 (17.76)	286.38 (12.21)	↑	0.72	n.a
	Choline	283.68 (24.50)	285.47 (38.52)	↑	0.87	n.a
	LDL_VLDL	156.25 (39.12)	171.78 (36.19)	↑	0.35	0.24
Amino acid metabolism	Glycine	217.75 (31.53)	196.29 (17.62)	↓	0.07	− 0.18
	Alanine	67.57 (20.08)	55.12 (17.37)	↓	0.14	− 0.32
	Glutamate	26.42 (8.87)	24.04 (4.51)	↓	0.43	n.a
	Valine	99.17 (12.03)	108.07 (15.70)	↑	0.13	n.a
	Leu_lle	139.10 (15.84)	152.31 (20.75)	↑	0.08	0.19
	Glutamine	83.90 (13.86)	85.18 (15.72)	↑	0.83	n.a
	Cre_Crn	36.30 (7.95)	38.34 (7.32)	↑	0.55	n.a
	Isoleucine	32.14 (3.41)	31.64 (3.86)	↓	0.75	n.a
	Glutamine	73.89 (13.72)	88.56 (14.04)	↑↑	0.0098**	0.23

The up or down arrow (↑/↓) indicates whether the metabolite showed a signal increase or decrease, respectively

The coefficient was calculated by Lasso regression. EA, migraine patients in the EA group after four weeks of EA treatment; Sham EA, migraine patients in the Sham EA group after four weeks of Sham EA treatment

*p < *0.05*

**p < *0.01*

### Functional Regulation and Network Adjustment by Acupuncture

After pinpointing the potential metabolic biomarkers for the clinical efficacy of acupuncture, we conducted a metabolic pathway and network analysis among the healthy control, MA, EA and Sham EA groups to further explore the potential upstream pathological mechanisms of acupuncture for migraine. By using IPA, we observed a significant change in the tRNA charging pathway (*p* = 0.031) and a top-changed metabolic network of carbohydrate metabolism, molecular transport, and small molecule biochemistry (score = 14) (Krämer et al. 2014) between the healthy control and MA groups (Fig. S3a, d).

After 20 sessions of EA treatment for four weeks, we found that the pyruvate fermentation to lactate pathway (*p* = 0.0175) was significantly reversed between the MA and EA groups and that the carbohydrate metabolism, energy production, and lipid metabolism networks (score = 9) were the top changed networks between the MA and EA groups (Fig. S3b, e). These results manifested that acupuncture reversed the levels of citrate and pyruvic acid through pyruvate fermentation to the lactate pathway, and changed the similar carbohydrate metabolism network, which was altered in migraine patients compared to healthy controls. The changed network of energy production in the EA group further supports the effect of EA based on regulations in energy metabolism from our previous results. Specifically, we found that the tRNA charging pathway (*p* = 0.0307) was significantly discriminated between the EA group and Sham EA group (Fig. S3c), and the top network discriminating the EA group and Sham EA group was carbohydrate metabolism, molecular transport, and small molecule biochemistry (score = 14, Fig. 4c). Glycerine was located near the center of the metabolic network of EA compared to Sham EA (Fig. 4c). These findings indicate that the metabolic difference found between EA and Sham EA treatment was linked to the tRNA charging pathway and carbohydrate metabolism, molecular transport, and small molecule biochemistry. These significantly changed metabolic pathways and networks may be the functional basis of the specific effects of EA compared with sham EA.

## Discussion

Here, we present the first metabolomic study of acupuncture for migraine derived from a randomized controlled trial. By using validated NMR techniques and well-designed machine learning strategies, we demonstrated that EA at acupoints could restore energy deficiency and adjust plasma citrate levels, thereby alleviating migraine. Using advanced Lasso regression models, we first identified eight metabolic biomarkers that can be attributed to the potential metabolic basis for specific effects of acupuncture compared to sham acupuncture. Taken together, these results demonstrated a distinct metabolic phenotype that can serve as a novel scientific explanation for the efficacy of acupuncture in the clinical setting.

The strengths of this study include concealed central randomization, practical treatment prescriptions, and a rigorous machine learning statistical strategy. Compared to other metabolomics studies on acupuncture, the use of central randomization minimizes selection bias, and the independence of the statistician also reduces detection bias, thus enhancing the reliability and validity of our results in a clinical context (Zhao et al. 2017a, b). Additionally, the treatment prescriptions for acupoints and non-acupoints were developed based on the consensus of experienced acupuncture professors and Western medicine neurologists, as well as insights from meta-analyses, high-quality acupuncture trials, and experimental studies on acupuncture for migraine relief (Vickers et al. 2012; Linde et al. 2005; Shi et al. 2010). To optimize the effect of acupuncture for migraine patients, acupuncture treatments were administered five times per week, in line with the recommendations of an updated IPD meta-analysis (Vickers et al. 2018).

Specifically, Lasso regression was employed to eliminate the multicollinearity between metabolites and the inter-metabolite relationships comprising the metabolic network (LeWitt et al. 2017; Menni et al. 2017). By using this shrinkage method, eight potential biomarkers distinguishing the acupuncture and sham acupuncture can be selected. Moreover, Box-Cox transformation was applied prior to ANOVA analysis, which could be a powerful procedure for adjusting skewed distributions and continuous variables, thereby improving accuracy of statistical results (Yu et al. 2022). Further, Bonferroni correction, one of the strictest multiple testing correction methods, was executed in the study. This method not only could control the false positive rate, but also pinpointed the validated biomarker for acupuncture (Sedgwick et al. 2014). Simply put, we presented a novel and practical machine learning strategy in a randomized acupuncture trial, which could set serve as a model for handling high-dimensional metabolomics data in acupuncture trials.

In this study, we found that EA was more clinically important to patients than sham EA, especially for reducing the intensity of pain in migraine patients. First, EA, not sham EA, could significantly alleviate these clinical symptoms in migraine. Second, the effect size of the VAS score for pain relief in the EA group was markedly higher than that in the Sham EA group (Table 1). The EA group showed an average decrease of 1.05 cm more in the total 10 cm VAS compared with the Sham EA group, and this decrease reached the 1 cm MID in the VAS scale (Thorlund et al. 2011), which accounted for 10.5% greater pain relief than Sham EA treatment at clinic. These results were further in agreement with a long-term randomized trial on acupuncture for migraines (Zhao et al. 2017a, b). In addition, the nonsignificant results observed in both acupuncture groups after treatment can be attributed to the limit of statistical significance for small sample sizes as well as the penetration needles in sham EA, which had already been analyzed in a large IPD meta-analysis (Vickers et al. 2012; Li et al. 2012).

**Fig. 5:**
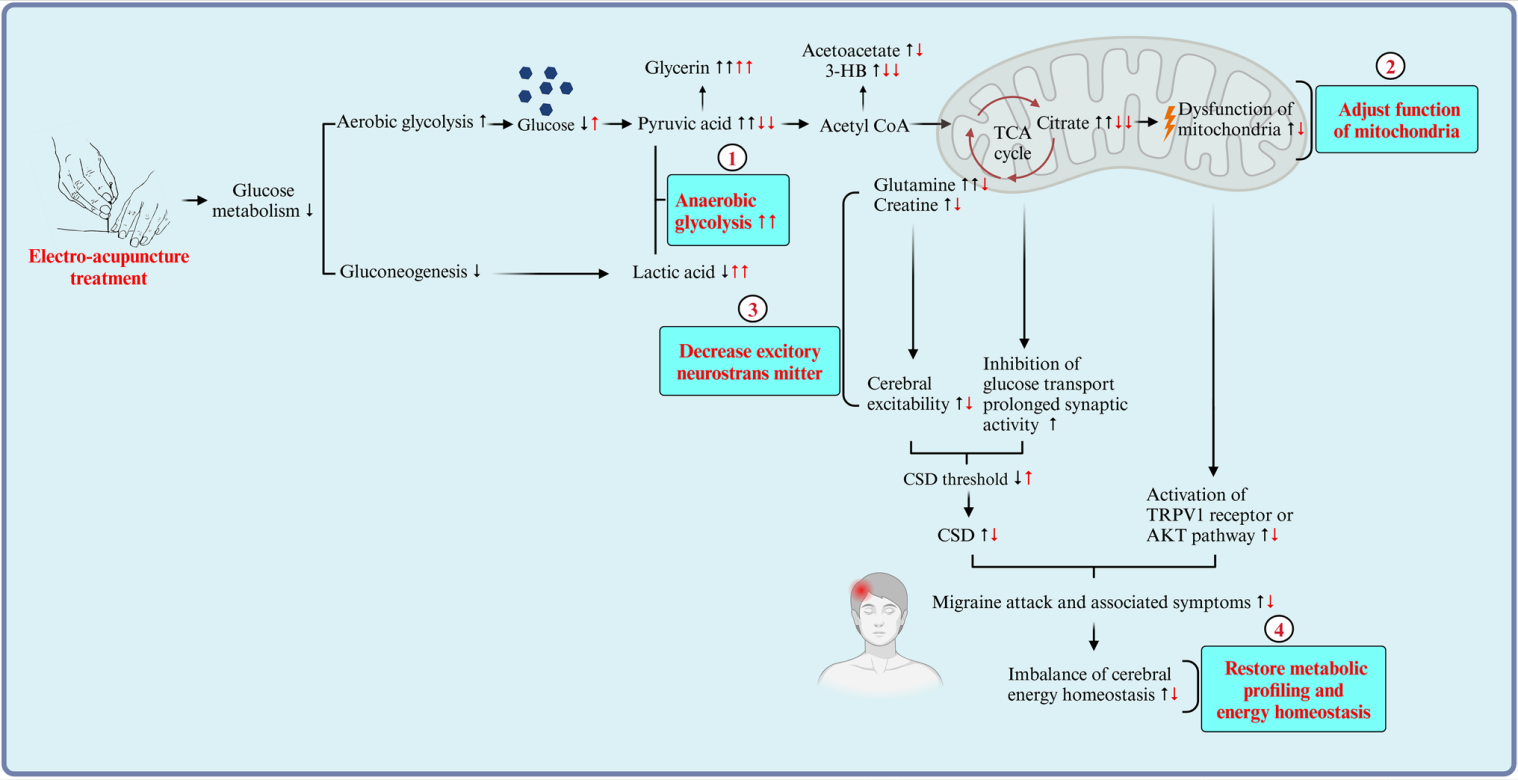
The metabolic mechanism for the efficacy of acupuncture in migraine. The black arrow (↑/↓) shows the depletion of glucose metabolism and increased lipid metabolism in migraine patients, leading to energy deficiency and disorder of the TCA cycle and mitochondria, which trigger migraine attack. The red arrow (↑/↓) and the blue box indicate the possible metabolic mechanism of acupuncture, which suggests that EA may restore energy deficiency by enhancing anaerobic glycolysis and lowering plasma citrate levels in the TCA cycle, thus decreasing migraine attack and restoring mitochondrial function and metabolic profiling in migraine patients

Recently, accumulating evidence has confirmed that energy deficiency and mitochondrial dysfunction are two cornerstones of migraine pathophysiology (Lodi et al. 2006; Sparaco et al. 2006). A novel metabolic picture of migraine has been presented in a publication of *Nature Reviews Neurology* (Gross et al. 2019): decreased cerebral glycogen prolonged synaptic activity, increased cerebral excitability, reduced CSD threshold, and thus stimulated CSD in migraine. On the other hand, mitochondrial dysfunction induced excessive production of free radicals and subsequently activated transient receptor potential (TRP) channels that increase CGRP release, which is pivotal for mediating migraine attack. Compared to previous studies, we demonstrated enhanced aerobic glycolysis and reduced gluconeogenesis, which trigger the increase in citrate as a key intermediate in the TCA cycle and lead to mitochondrial energy metabolism disorder in migraine patients. Lipid metabolism is also affected and disordered, accompanied by an increase in fat mobilization, glycerine and ketone bodies (Table 2, Fig. 5) (Aurora et al. 2007; Bélanger et al. 2011). These findings further detailed the imbalance between reduced energy supplies and increased energy needs among migraine patients (Fig. 5). In addition, we demonstrated that citrate performed well in discriminating migraine patients and healthy controls, revealing a potential novel biomarker for migraine. It has been shown that an increase in citrate could activate transient receptor potential vanilloid 1 (TRPV1) pain receptors, enhance the protein kinase B (AKT) signaling pathway (Fig. S3d) (Gross et al. 2019; Liu et al. 2017; Xu et al. 2018; Chen et al. 2017), thereby trigger migraine (Table 2, Fig. 5). Collectively, our findings not only expanded the pathophysiology of migraines with an additional understanding of metabolism, but also highlighted the demands of efficient metabolic strategies for migraine treatment.

Previously, there was little understanding of the metabolic basis of acupuncture in relieving migraine. Here, we provid novel metabolic evidence of EA relieving migraine as follows: (i) EA enhances anaerobic glycolysis through converting increased pyruvic acid into lactic acid and subsequently decreases elevated level of citrate in the TCA cycle and acetoacetate in lipid metabolism in migraine patients (Fig. 5). (ii) Importantly, citrate, which was found to be significantly increased in migraine patients, was significantly decreased after EA treatment, providing direct metabolic evidence that EA adjusts mitochondrial function in migraine (Table 2, Fig. 5). To date, both experimental and clinical evidence has shown that the depletion of glycogen and hypoglycaemia can trigger migraine attacks (Lodi et al. 2006; Pearce et al. 1971; Hockaday et al. 1971). And lactic acid is a prior energy source for neurons during brain energy deficiency (Suzuki et al. 2011). Therefore, the first possible metabolic mechanism by which EA relieves migraines is the increased anaerobic glycolysis caused by EA combined with the increase in lactic acid, which can rapidly compensate for energy deficits and thereby possibly reduce the CSD triggering migraines (Fig. 5). Similarly, a positron emission tomography–computed tomography (PET-CT) study (Zeng et al. 2012; Yang et al. 2012) also revealed that EA could restore glucose metabolism in key regions of the descending pain modulation system (DPMS) of brain for migraine. Interestingly, our findings identified a potential subgroup of migraine patients with a distinct metabolic phenotype that could benefit from EA treatment in clinical settings. Moreover, previous studies have highlighted an important role of mitochondrial dysfunction in migraine and the positive association between citrate levels and activation of the AKT signaling pathway (Gross et al. 2019; Liu et al. 2017; Xu et al. 2018; Chen et al. 2017). Recent animal experiment further demonstrated inhibition of the activation of AKT could attenuate cumulative pain score and pain-related behaviors (Xu et al. 2019). Thus, the second potential metabolic mechanism of EA managing migraine is that EA could adjust mitochondrial dysfunction and inhibit the pain-related AKT pathway by decreasing the plasma level of citrate, thereby alleviating pain in migraine (Fig. 5). In particular, this metabolic biomarker of citrate may serve as the desired quantitative biomarker for further clinical acupuncture research. Third, in the previous decades, profound acupuncture experiments focused on the pivotal role of opioid peptides and its related arcuate nucleus-periaqueductal gray-nucleus raphe magnus (Arc-PAG-NRM)-spinal dorsal horn pathway in mediating acupuncture analgesia (Zhao et al. 2008; Huang et al. 2002). Compared to the neurological evidence of acupuncture analgesia, our results revealed a systemic modulatory effect of EA on both energy metabolism and mitochondrial function in migraine patients. These findings revealed exceptional new insights into the metabolic mechanism underlying the effectiveness of acupuncture analgesia and could lead to the development of potent non-opioid drugs and multi-metabolic therapeutic targets for future migraine treatment.

The specific effect of acupuncture is another long-debated issue in clinical acupuncture trials. In 2005, a multi-center acupuncture trial treating migraine published in JAMA sparked the debate over the difference of effectiveness between acupuncture and sham acupuncture (Linde et al. 2005). Following this trial, an individual patient data (IPD) meta-analysis involving 20,827 patients, also published in JAMA, confirmed that acupuncture is statistically superior to sham acupuncture for migraines, although the difference in effect size was relatively small (Vickers et al. 2012; Vickers et al. 2014). In 2017, Zhao et al. (2019) further demonstrated that acupuncture for migraine is more effective than sham acupuncture in a multicenter randomized trial published in JAMA internal medicine. In 2022, Fei et al. (2022) and Prof. Gordon Guyatt further highlighted in BMJ the methodological challenges in acupuncture trials, particularly in estimating the optimal treatment effect of acupuncture compared to sham acupuncture. Based on the current methodology challenges in acupuncture trials, we present a unique metabolic mechanism for the specific effect of acupuncture: We demonstrate that there are important but different ways of energy supply between EA and sham EA for relieving migraine. (i) EA specifically reverses the deficiency of energy metabolism in migraine patients through anaerobic glycolysis compared with Sham EA. In detail, EA can restore energy deficiency by enhancing anaerobic glycolysis and the decomposition of acetoacetate (Table 2, Fig. 5) in the plasma (Salek et al. 2017; Peek et al. 2020), which could quickly compensate for energy deficits and thereby reduce the CSD triggering migraine. In contrast, sham EA may partially supply energy to migraine patients by fat mobilization through lipid metabolism (Table 3). Additionally, network analysis showed that glycerine, a classic product of fat mobilization, was the key node in the metabolic network of EA compared with sham EA (Fig. 4c). Therefore, we can postulate the hypothesis that these different ways of supplying energy may be crucial factors contributing to the differences in effect between acupuncture and sham acupuncture. (ii) EA specifically relieves migraine by reversing the level of citrate, thereby adjusting mitochondrial dysfunction compared with sham EA. The TCA cycle is the center of energy metabolism in the body. Specifically, we have shown that EA, not sham EA, could reverse plasma citrate and subsequently adjust the dysfunction of the TCA cycle in mitochondria, thus restoring metabolic profiles and relieving migraine in migraine patients (Fig. 5). (iii) We found that glutamine, a classic excitatory neurotransmitter that facilitates CSD and is linked to the intensity of migraine (Gao et al. 2014; Aroke et al. 2020; Alam et al. 1998), showed a Bonferroni-corrected significant decrease in the EA group compared with the Sham EA group. This result is consistent with our previous metabolomic study of an acute migraine rat model (Gao et al. 2014). The significant decrease in glutamine in the EA group compared to the Sham EA group further explains why EA was more effective for clinical migraine pain relief than sham EA. Additionally, our study found that both EA and sham EA significantly increase glycerine levels, thereby supplying energy to migraine patients. This finding gives a possible explanation for the non-specific effect of EA and sham EA observed in these clinical trials. Collectively, the specific effect of EA on both anaerobic glycolysis and mitochondrial function for migraine exclusively provides a possible scientific mechanism for the efficacy of acupuncture for clinical doctors and clinical decision-makings. The discovery of distinct energy supply mechanisms contributing to different effects between acupuncture and sham acupuncture may offer a unique metabolic mechanism to address the methodology challenges of estimating the specific effect of acupuncture over the past decade.

The main limitation of this study is the limited sample size and the non-targeted metabolic technique, which cannot detect targeted metabolite changes in the TCA cycle and glucose metabolism. Additionally, validated animal experiment should also be conducted. Future studies will extend this work using a larger sample size of migraine patients and targeted metabolomics in both human and animals for possible validation experiments.

## Conclusions

In summary, our findings indicate that EA specifically relieves migraine by enhancing anaerobic glycolysis and decreasing the plasma levels of citrate and glutamine among migraine patients. Our study is of great importance because it provides scientific evidence and explanations for the specific effect of acupuncture relieving migraine for both clinical doctors and policymakers. By applying modern machine learning techniques, this metabolic evidence could enlighten a brand new direction into acupuncture analgesia mechanism, which in turn would pose new challenges for future acupuncture researches.

## Supplementary Information

Below is the link to the electronic supplementary material.

Supplementary file1 (DOCX 1988 KB)

## Data Availability

All data, models, or code generated or used during the study are available from the corresponding authors on reasonable request.

## Abbreviations

AKT: Protein kinase B
BMI: Body mass index
CGRP: Calcitonin gene-related peptide
CPMG: Carr-Purcell-Meiboom-Gill
CSD: Cortical spreading depression
DPMS: Descending pain modulation system
EA: True electroacupuncture
FFT: Fast Fourier Transform
GCP: Good Clinical Practice
3-HB: 3-Hydroxybutyric acid
HMDB: The Human Metabolome Database
IPA: Ingenuity Pathway Analysis
IPD: Individual patient data
ITT: Intention-to-treat
Lasso: Least absolute shrinkage and selection operator
LED: Longitudinal eddy current delay
MID: Minimal important difference
NAc: N-acetyl glycoproteins
NMR: Nuclear magnetic resonance
Leu_lle: Isoleucine
LDL_VLDL: Low density lipoprotein_very low density lipoprotein
OPLS-DA: Orthogonal signal correction for partial least square discriminate analysis
PET-CT: Positron emission tomography–computed tomography
Ptdcho: Phosphatidylcholine
Sham EA: Sham electroacupuncture
TCA: Tricarboxylic acid cycle
TCM: Traditional Chinese Medicine
TRP: Transient receptor potential
TRPV1: Transient receptor potential vanilloid 1
TSA: Total spectrum area
VAS: Visual analogue scale
